# The two pore channel TPC2 is dispensable in pancreatic β-cells for normal Ca^2+^ dynamics and insulin secretion

**DOI:** 10.1016/j.ceca.2015.12.004

**Published:** 2016-01

**Authors:** Matthew C. Cane, John Parrington, Patrik Rorsman, Antony Galione, Guy A. Rutter

**Affiliations:** a Section of Cell Biology and Functional Genomics, Imperial College London, Du Cane Road, W12 0NN London, UK; b Department of Pharmacology, University of Oxford, Mansfield Road, OX1 3QT, UK; c The Oxford Centre for Diabetes, Endocrinology and Metabolism, Churchill Hospital, Oxford OX3 7LJ, UK

**Keywords:** AUC, area under the curve, [Ca^2+]^_i_, intracellular free Ca^2+^ ion concentration, GLP-1, glucagon-like peptide-1, IPGTT, OGTT, intraperitoneal and oral glucose tolerance test, NAADP, nicotinic acid adenine dinucleotide phosphate, TPC1, TPC2, two pore channels 1 and 2, β-Cell, Calcium, TPC2, Diabetes, GLP-1, NAADP

## Abstract

Ca^2+^ signals are central to the stimulation of insulin secretion from pancreatic β-cells by glucose and other agents, including glucagon-like peptide-1 (GLP-1). Whilst Ca^2+^ influx through voltage-gated Ca^2+^ channels on the plasma membrane is a key trigger for glucose-stimulated secretion, mobilisation of Ca^2+^ from acidic stores has been implicated in the control of more localised Ca^2+^ changes and membrane potential. Nicotinic acid adenine dinucleotide phosphate (NAADP), generated in β-cells in response to high glucose, is a potent mobiliser of these stores, and has been proposed to act through two pore channels (TPC1 and TPC2, murine gene names *Tpcn1* and *Tpcn2*). Whilst the role of TPC1 in the control of Ca^2+^ mobilisation and insulin secretion was recently confirmed, conflicting data exist for TPC2. Here, we used the selective and efficient deleter strain, Ins1*Cre* to achieve β-cell selective deletion of the *Tpcn2* gene in mice. βTpcn2 KO mice displayed normal intraperitoneal and oral glucose tolerance, and glucose-stimulated Ca^2+^ dynamics and insulin secretion from islets were similarly normal. GLP-1-induced Ca^2+^ increases involved an increase in oscillation frequency from 4.35 to 4.84 per minute (*p* = 0.04) at 8 mM glucose, and this increase was unaffected by the absence of *Tpcn2*. The current data thus indicate that TPC2 is not absolutely required for normal glucose- or incretin-stimulated insulin secretion from the β-cell. Our findings suggest that TPC1, whose expression tended to increase in *Tpcn2* null islets, might be sufficient to support normal Ca^2+^ dynamics in response to stimulation by nutrients or incretins.

## Highlights

• Mice null for *Tpcn2* in pancreatic β-cells were created using *Lox*P-mediated deletion.
• The absence of TPC2 had no impact on intraperitoneal or oral glucose tolerance.
• Glucose and GLP-1-induced Ca^2+^ dynamics were unaffected in null mouse islets.
• Functional redundancy of TPC channels appears to exist in β-cells.

## Graphical abstract

**Figure:**
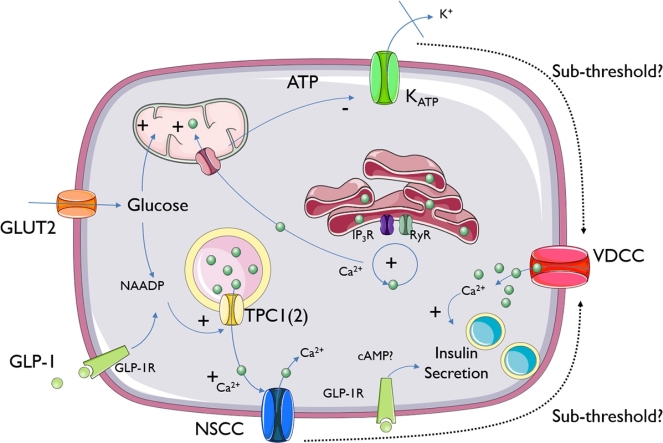

## 1 Introduction

The pancreatic islet β-cell is an important model for the study of fuel sensing and stimulus-secretion coupling [1], [2]. Derangement of normal β-cell function [3], as well as a decrease in overall β-cell mass [4], [5], underlie insulin deficiency in type 2 diabetes [1], [6], [7] a disease which now affects more than 8% of the adult population worldwide [8].

Classically, elevated blood glucose levels are believed to stimulate insulin secretion through an increased cytosolic ATP:ADP ratio [9], [10] leading to closure of ATP-sensitive K^+^ channels (K_ATP_) [11] and enhanced Ca^2+^ influx through voltage-dependent Ca^2+^ channels (VDCCs). Whilst substantial pharmacological [11] and genetic [12], [13] evidence supports this model, it is undoubtedly incomplete, not least because deletion of K_ATP_ channel subunits (SUR1/*ABCC8* and Kir6.2/*KCNJ11*) does not render β-cells wholly glucose-unresponsive [14], [15]. Indeed, it is increasingly accepted that an integration of nutrient, neurotransmitter and hormonal signals is necessary for the induction of insulin release *in vivo* [1], [2].

Internal Ca^2+^ stores, including those associated with the endoplasmic reticulum (ER) [16], and acidic stores, are also known to modulate intracellular free Ca^2+^ ([Ca^2+^]_i_) signals in β-cells [2]. Of note, alkalinisation of acidic stores by inhibition of the V_o_-ATPase with Bafilomycin A1 impedes glucose-induced [Ca^2+^]_i_ responses in these cells [17], [18]. Nicotinic acid adenine dinucleotide (NAADP) is an important second messenger which has been implicated in the recruitment of acidic stores for the generation of [Ca^2+^]_i_ signals. Produced by the endosomal/cell surface-located enzyme CD38 *via* a base exchange reaction [19], NAADP is generated in β-cells in response to glucose [20], and the incretin hormone glucagon-like peptide-1 (GLP-1) [21]. Whilst the latter G-protein receptor-coupled hormone potently stimulates insulin secretion at permissive glucose concentrations, allowing the development of incretin-based therapies for type 2 diabetes [22], [23], [24], the impact of GLP-1 on β-cell Ca^2+^ dynamics is less well established and appears to be species dependent [25], [26], [27].

Whilst also a matter of debate, consensus is building that one or more of the two pore channel subtypes (TPC) serves as the putative NAADP receptor Ca^2+^-release channel. Nevertheless, it is also possible that TPCs form one part of a channel complex that also includes a distinct NAADP-binding protein. Expressed on endo-lysosomal compartments, TPCs (gene name *TPCN*) have been implicated in the generation of NAADP-mediated Ca^2+^ signals in β- and other cells. Thus, TPC2 expression renders NAADP-unresponsive cells responsive [28], and expression of cell surface targeted TPCs allows NAADP to induce Ca^2+^ influx [29]. Furthermore, both the rodent *Tpcn2* and the orthologous human *TPCN2* gene have been identified as potential causal genes for diabetes-associated traits [30].

Despite this body of data indicating an important role of TPC2 in Ca^2+^ signalling in β-cells, knock out of the *Tpcn2* gene in various animal models has shown divergent effects. For example, global deletion of the *Tpcn2* gene in the mouse through the use of a gene trap vector renders pancreatic β-cells unresponsive to NAADP either through use of the cell permeable analogue NAADP-AM or through introduction of NAADP directly through the patch pipette [17], [28]. Similarly, glucose-induced Ca^2+^ signals are also somewhat impaired in *Tpcn2*^−/−^ mice, although they are not fully ablated [17]. Nonetheless, glucose-induced insulin secretion was barely affected and glucose tolerance was not abnormal in *Tpcn2*^*−/−*^ animals [17]. On the other hand, *Tpcn1*^−/−^ mice showed clear differences in both parameters [17]. Of note, although the *Tpcn2*^−/−^ mice used in a recent study by Wang et al. [18] similarly failed to show any abnormalities in terms of responses to NAADP, the truncation in this allele may still enable the expression of a functional TPC2 protein [31].

In light of these discrepancies, we have generated β-cell-specific *Tpcn2* knockout mice by crossing animals harbouring a *flox*ed exon of the *Tpcn2* gene to knock-in mice expressing *Cre* recombinase at the endogenous *Ins1* locus [32], [33]. This strategy results in efficient (∼95%) recombination in β-cells [32], [33] (Johnston et al, unpublished results) throughout the islet. Furthermore, and in contrast to other currently-available insulin promoter-driven *Cres*, notably RIP2Cre [34], [35], [36], recombination with the Ins1*Cre* strain is not complicated either by off-target events including recombination in the brain [37] nor by the simultaneous ectopic expression of human growth hormone (hGH) in the β-cell.

This approach has enabled us to study further the role of TPC2 in the β-cell whilst eliminating confounding effects which may result from the deletion of the *Tpcn2* gene in other tissues. After confirming ablation of *Tpcn2* expression we have used this model to determine the cell autonomous role of TPC2 in the β-cell, focussing on glucose homeostasis, insulin secretion and the regulation of Ca^2+^ dynamics by glucose and incretins.

## 2 Methods

### 2.1 Animal origin and maintenance

Mice heterozygous for the *flox*ed allele of the *Tpcn2* gene (exon 6 flanked by *lox*P sites) were obtained from the European conditional mouse mutagenesis program (EUCOMM) *via* MRC Harwell, U.K. Mice bore the “Tm1c” (http://www.mousephenotype.org/about-ikmc/eucomm-program/eucomm-targeting-strategies)allele (Tpcn2^tm1c(EUCOMM)Hmgu^) and were crossed with Ins1Cre-expressing animals [32]. The subsequent litters were back-crossed to generate Tpcn2^*fl*/*fl*^:Ins1Cre^+/−^ (referred to as ‘KO’) and Tpcn2^*fl*/*fl*^:Ins1Cre^−/−^ (referred to as ‘control’) mice. Offspring were bred together to generate litters with ∼50% WT mice and ∼50% β-cell specific *Tpcn2*-deleted mice. Animals bearing the Ins1Cre transgene alone displayed no abnormalities in glucose tolerance, insulin secretion or β-cell mass [32].

Animals were maintained on a C57/BL6 background and 2 to 4 mice were housed per individually-ventilated cage in a pathogen-free facility. Mice were kept under a 12 h light/dark cycle and fed a regular chow diet (Lillico Biotechnology). All *in vivo* experiments were performed on male mice and islets were isolated from an equal number of male and female mice. All animal experiments were approved by the UK Home Office under the Animals (Scientific Procedures) Act 1986 (PPL 70/7349).

### 2.2 qRT-PCR

Approximately 100 freshly isolated islets were used for RNA extraction using TRIzol reagent (Invitrogen) and cDNA was generated using a high capacity reverse transcription kit (Applied Biosystems) according to the manufacturer's instructions. SYBR Green qRT-PCR was performed as previously described (REF) and β-Actin was used as the reference gene. See Table 1 for list of primers.

### 2.3 Glucose tolerance tests

Mice were fasted overnight before i.p. injection or oral gavage of 30% glucose in water to a dose of either 1 g/kg or 3 g/kg. Blood was sampled from the tail vein at 0, 15, 30, 60 and 120 min and glycaemia measured using an automatic glucometer (Accu-chek, Roche).

### 2.4 Insulin secretion

Islets were isolated by collagenase dissociation as described previously [38] and ∼50 islets were perifused with Krebs-HEPES buffer (in mM, 130 NaCl, 3.6 KCl, 0.5 NaH_2_PO_4_, 2 NaHCO_3_, 1.5 CaCl_2_, 1.2 MgCl_2_, 3 D-glucose, 10 HEPES, 0.5 MgSO_4_, pH 7.4 substituted with 0.1% bovine serum albumin) at a rate of 500 μl min^−1^ at 37 °C in a custom-built chamber. Perifusate was collected in 30 s intervals. Islets were primed with Krebs medium containing 3 mM glucose for 30 min prior to the experiment and glucose was increased to 16.9 mM 2 min after the initial collection. Islets were then frozen in 10 ml acidic ethanol (1.5% 1 M HCl, 75% EtOH, 0.1% Triton X-100) until assayed for total insulin content. The latter samples were diluted 1:100 whilst secreted insulin samples were assayed undiluted using an HTRF insulin assay kit (Cisbio assays) and a PHERAstar reader (BMG), following the manufacturer's guidelines.

### 2.5 Calcium imaging

Experiments were performed essentially as previously described [26]. In brief, islets were loaded with 10 μM Fluo-8 AM (Abcam) dissolved in DMSO (0.01% wt/vol) in Krebs-HEPES buffer for 45 min at 37 °C. Islets were visualised using a Zeiss Axiovert confocal microscope and perifused continuously at 34–36 °C with Krebs-HEPES buffer containing relevant concentrations of glucose and GLP-1. Fluo-8 was excited with a 491 nm laser and emitted light collected at 525 nm. Volocity™ software (Perkin–Elmer) was used for imaging and analysis. Traces were normalised to the initial fluorescence (*F*/*F*_0_).

### 2.6 Statistics

Data were analysed using GraphPad PRISM 6.0 software and significance was tested using unpaired Student's two-tailed *t*-tests with Bonferroni post-tests for multiple comparisons, or two-way ANOVA as indicated. *p* < 0.05 was considered significant and errors signify mean ± SEM.

## 3 Results

### 3.1 Unaltered glucose tolerance after targeted deletion of Tpcn2 from β-cells

**Fig. 1:**
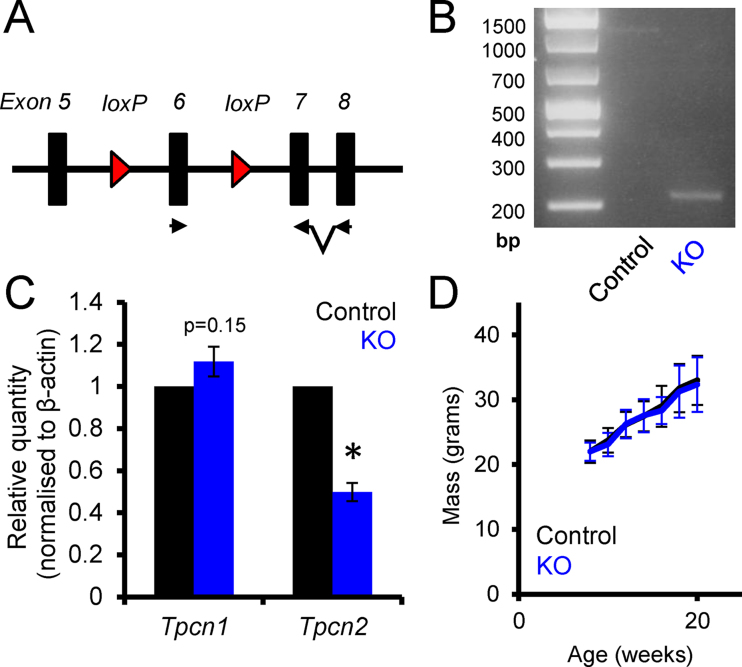
(A) Schematic of the *flox*ed allele of *Tpcn2* showing the location of *loxP* sites flanking exon 6 and the target sites of primers used in (C). (B) The excision event resulting from the cross with Ins1Cre mice as demonstrated by the removal of the critical region containing exon 6. (C) Relative expression of *Tpcn2* in control (black) and β-cell-specific *Tpcn2* KO (blue) islets, normalised to β-actin (*Actb*). *p < 0.05 for the effect of *Tpcn2* deletion. (D) Changes in the weight of control (black) and KO (blue) animals from 8 to 20 weeks of age. (For interpretation of the references to colour in this figure legend, the reader is referred to the web version of this article.) See Table 1 for details of primers used for PCR and qRT-PCR.

To delete TPC2 specifically from the pancreatic β-cell, mice bearing alleles of the *Tpcn2* gene with *lox*P sites flanking exon 6 were crossed to mice bearing the Ins1*Cre* transgene [32] (Fig. 1A). In the first instance, we confirmed the *Cre* excision event by PCR. Primers flanking the *lox*P sites were expected to generate a 1055 bp fragment in the control islets, and a smaller fragment of 215 bps in the KO islets, after the excision event has taken place. The corresponding bands can be seen in Fig. 1B. Secondly, we confirmed knock down of the expression of *Tpcn2* by qRT-PCR. Relative expression of the Tpcn2 transcript from KO islets was decreased to ∼50% of control levels. This reduction is compatible with essentially complete deletion from β-cells, assuming a β-: α-cell ratio of ∼3:1 [39] and a ratio of *Tpcn2* mRNA of 1:3 in β-: α-cells [40]. *Tpcn1* mRNA levels showed a slight tendency towards an increase in *Tpcn2* null islets (*p* = 0.147, Fig. 1C).

**Fig. 2:**
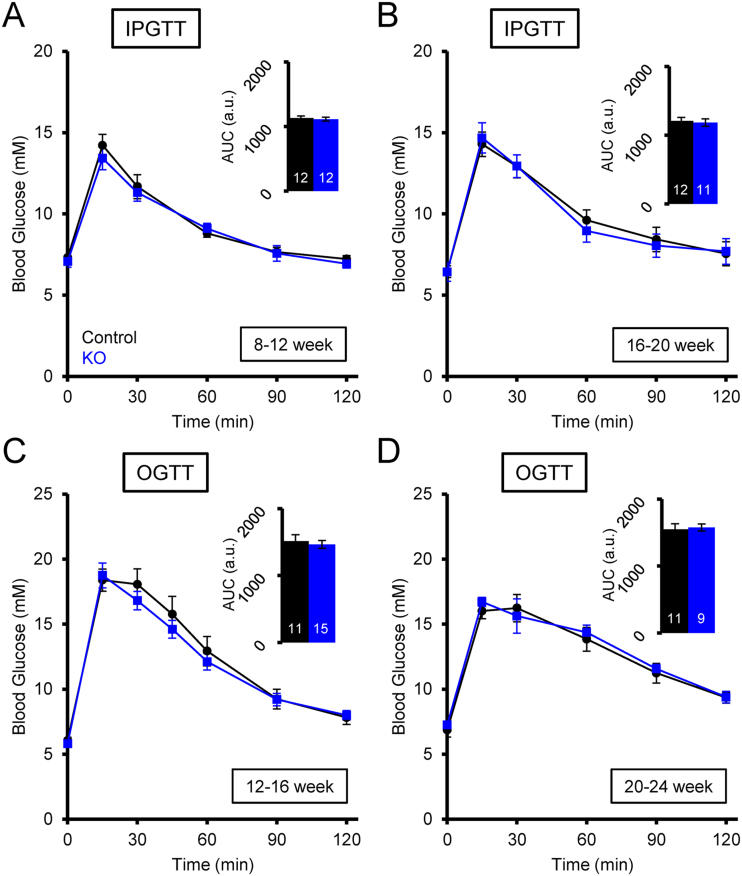
Glucose tolerance tests of control and β*Tpcn2* KO mice. Glucose tolerance after intraperitoneal (ip) glucose injection (A, B) or oral glucose *via* gavage (C, D) of male mice. Mice were given 1 g/kg glucose with the exception of the 12–16-week OGTT mice (C) which were given a higher dose of 3 g/kg. Experiments were quantified by area under the curve (AUC) analysis as shown in the inset bar graphs and the number of mice used in each experiment is shown in white. Data are expressed as means ± SEM. No statistically significant differences were observed.

β*Tpcn2* KO mice were indistinguishable from littermate control animals by visual inspection, and displayed normal growth and weight changes (Fig. 1D). Glucose tolerance was investigated in these mice by both intraperitoneal (IPGTT) and oral (OGTT) administration of the sugar, the latter allowing exploration of the incretin effect on insulin secretion. Mice underwent IPGTTs at 8–12 weeks, and then again at 16–20 weeks of age by administration of 1 g/kg glucose. Whilst a small decrease in glucose tolerance was observed between the younger and older mice, no significant differences were observed between the β*Tpcn2* KO mice and their littermate controls in either age group (Fig. 2A, B). Similarly, no significant difference between genotypes was observed during either OGTT at 12–16 weeks (performed using a higher concentration of 3 g/kg glucose) or at 20–24 weeks of age (Fig. 2C, D).

### 3.2 Normal glucose-induced Ca^2+^ dynamics and insulin secretion in βTpcn2 KO mouse islets

**Fig. 3:**
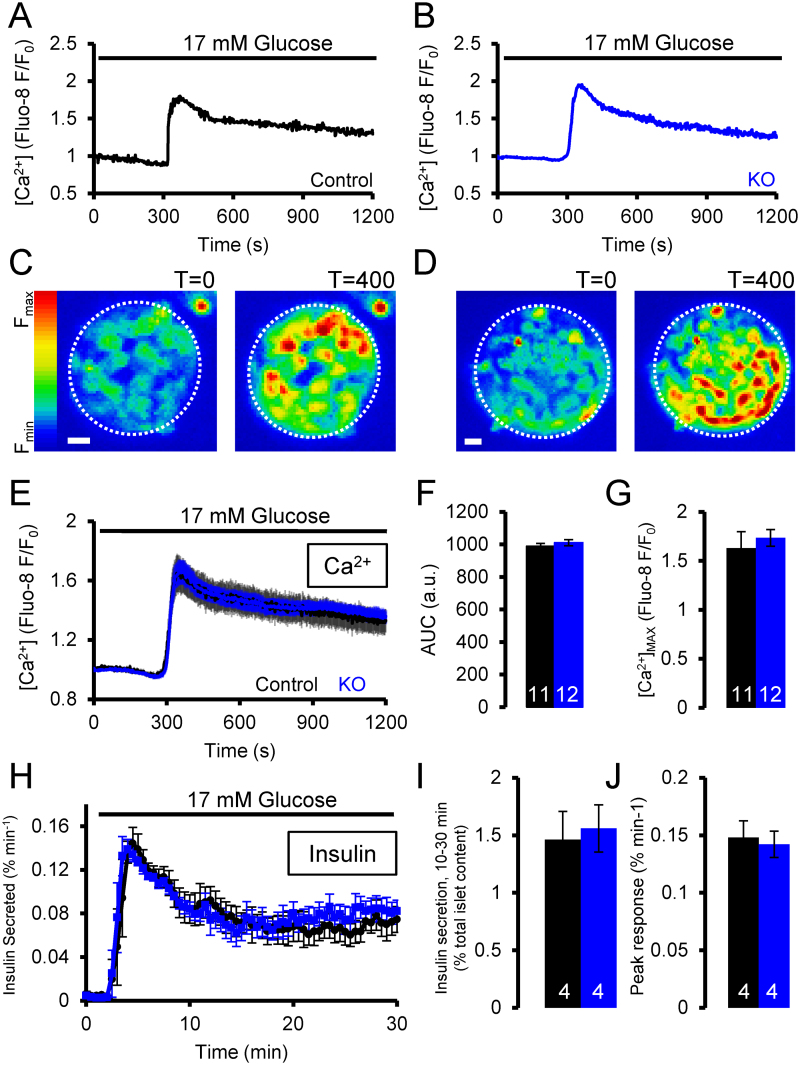
Glucose responsiveness of control and β*Tpcn2* KO islets. (A–G) Ca^2+^ responses to 17 mM glucose in control islets (blue) and β-cell specific *Tpcn2* KO islets (black). Example Ca^2+^ responses (A, B), and corresponding images of basal and peak fluorescence showing the global response over the whole islet (C, D) where scale bars correspond to 20 μm. (E) Averaged response showing the mean ± SEM of Ca^2+^ traces. These data are quantified by area under curve analysis (F) and the average peak fluorescence (G). (H–J) Insulin secretion from isolated islets from control (black) and beta cell specific *Tpcn2* knock-out mice (blue). (H) Insulin secreted over time shown as mean ± SEM. Quantification of the insulin secretion during the period from 2 to 32 min after the start of the experiment by insulin secreted as a percentage of the total insulin content of the islets (I) and peak insulin response (J). The number of islets used for each set of experiments is shown on the bars in white. (For interpretation of the references to colour in this figure legend, the reader is referred to the web version of this article.)

Having demonstrated a lack of any effect of β-cell-selective TPC2 ablation on glucose tolerance *in vivo*, where compensatory effects might conceivably obscure changes in β-cell glucose responsiveness, we performed studies using islets *ex vivo* to determine whether a more subtle role of TPC2 deletion might be unmasked. The effect of TPC2 deletion in β-cells on Ca^2+^ signals was investigated by live fluorescence microscopy on isolated islets loaded with the Ca^2+^ indicator Fluo-8. Peak-plateau responses in intracellular free Ca^2+^ ([Ca^2+^]_i_) were generated in whole islets by the addition of high (17 mM) glucose after a period of exposure to low (3 mM) glucose. Regions of interest were drawn around the whole islet to observe the coordinated response to increased glucose (Fig. 3 A–G). TPC2 deletion had no obvious effect on the above Ca^2+^ responses, after analysis of both the peak and the area under curve (AUC). Likewise, changes in membrane potential after high glucose treatment were similarly not affected by the absence of TPC2 (Sones, W and PR, results not shown, *n* = 3).

Insulin secretion from isolated islets was next monitored dynamically over time using a custom built perifusion apparatus. Insulin secretion was induced by the addition of 17 mM glucose after a period of exposure to low glucose (3 mM). Insulin secretion began immediately after the increase in glucose concentration and achieved a peak rate of secretion of approximately 0.14% of the total insulin content per minute. Secretion then slowed to a plateau around 0.07% of total insulin per minute. The absence of TPC2 from β-cells resulted in no evident change in either the peak rate of this response nor in the overall insulin secreted, as represented by analysis of the area under the curve (Fig. 3 H–J).

### 3.3 Normal incretin-induced Ca^2+^ dynamics and insulin secretion in βTpcn2 KO mouse islets

**Fig. 4:**
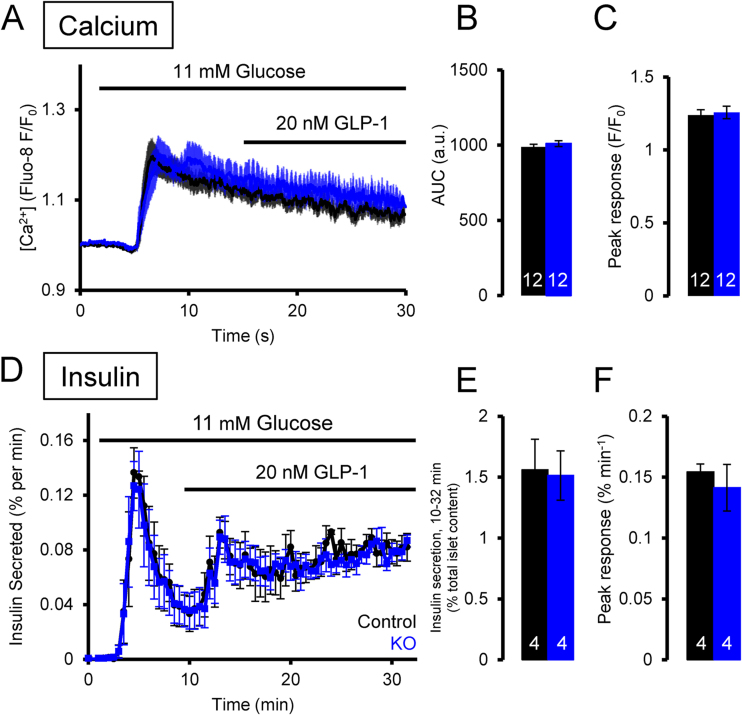
Effect of GLP-1 on control and β*Tpcn2* KO islets (A) Average Ca^2+^ responses showing mean ± SEM. (B, C) Quantification of the area under the curve (AUC) and peak of Ca^2+^ traces. (D) Insulin secretion from isolated islets from control (black) and β*Tpcn2* KO mice (blue). (E, F) Quantification of insulin secretion during the period from 2 to 32 min after the start of the experiment for the 17 mM glucose-stimulated islets, or from 10 to 32 min for the 11 mM glucose plus GLP-1-stimulated islets. The number of islets used for each set of experiments is shown on the bars in white. (For interpretation of the references to colour in this figure legend, the reader is referred to the web version of this article.)

We next used the same techniques to determine whether TPC2 may contribute to incretin-regulated Ca^2+^ dynamics. Islets were stimulated first with 11 mM glucose and additionally with 20 nM GLP-1 eight minutes later. Addition of the incretin at this concentration of glucose did not lead to any significant effect on [Ca^2+^]_i_ in control or KO islets (Fig. 4 A–C). However, and despite not significantly affecting Ca^2+^ signals, GLP-1 did induce a rapid and significant increase in the plateau of insulin secretion (Fig. 4 D–F), indicating that the ability of GLP-1 to increase insulin release was *via* potentiation of Ca^2+^-dependent exocytosis but did not result from an increase in [Ca^2+^]_i_ beyond that produced by glucose alone. TPC2 ablation did not affect the secretory response to GLP-1, nor insulin secretion in response to 8 mM glucose alone, as confirmed by area under the curve analysis and measurement of peak responses.

### 3.4 Subtle changes in GLP-1-induced Ca^2+^ transients during moderate glucose stimulation of βTpcn2 KO islets are indistinguishable from control

**Fig. 5:**
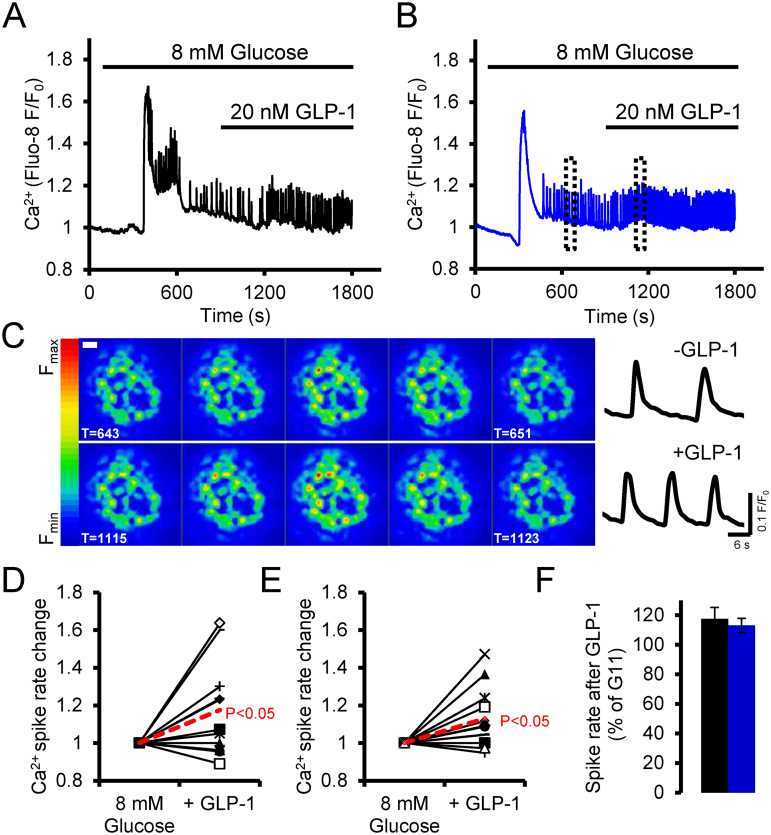
Effect of GLP-1 on Ca^2+^ oscillations in control and β*Tpcn2* KO islets. (A, B) Example traces of control islets (blue) and β-cell-specific *Tpcn2* KO islets (black), showing Ca^2+^ responses to 8 mM glucose with the addition of GLP-1. Boxes in (B) indicate regions shown in (C). (C) Example Ca^2+^ oscillations before and after the addition of GLP-1 after the 8 mM glucose response in a KO islet. The Ca^2+^ signal, as observed during a single oscillation (image capture at 2 s intervals) is apparent across the whole islet. The increase in oscillation frequency is apparent between the upper (−GLP-1) and lower (+GLP-1) traces. Scale bar = 20 μm. (D, E) Change in oscillation frequency upon GLP-1 stimulation normalised to the rate induced by 8 mM glucose. Oscillations were counted in a 5 min window before and after GLP-1 addition and the red dotted line indicated the average increase. *p* < 0.05 for the effects of GLP-1 in each case. (F) Quantification of the increase in oscillation frequency, showing no significant difference between control and β*Tpcn2* KO islets (mean ± SEM). (For interpretation of the references to colour in this figure legend, the reader is referred to the web version of this article.)

In an attempt to investigate a more subtle role of GLP-1 in Ca^2+^ signalling and subsequently to investigate the role of TPC2 in this response, we induced stable Ca^2+^ oscillations in isolated mouse islets by stimulating with 8 mM glucose. The resulting oscillations were variable between islets as can be seen in the individual example traces (Fig. 5A, B). The oscillations were also systemic across the islet and not specific to individual cells or regions (Fig. 5C). The addition of GLP-1 after glucose administration increased the frequency of these oscillations without prompting any recruitment of previously quiescent cells into activity (Fig. 5 A–C). Islets showed varying responses to GLP-1 but a significant number showed an increase, as can be seen by the increase in frequency shown in (Fig. 5 D–F). However, no effect of *Tpcn2* deletion on this modulation of Ca^2+^ oscillation frequency was observed.

## 4 Discussion

### 4.1 Role of TPC2 in the beta cell

We describe here the impact on glucose homeostasis and Ca^2+^ dynamics of β-cell selective deletion of *Tpcn2* in the mouse. Elimination of TPC2 was confirmed by demonstrating a reduction in transcript level compatible with a selective loss from the β-cell compartment, as expected with the use of the efficient and β-cell-selective Ins1*Cre* strain [32]. Confirmation of knockdown at the protein level was not possible since commercially available antibodies proved to be non-specific in our hands (results not shown).

The use of pharmacological agents (*e.g.* Bafilomycin A1) to alkalinise this compartment, or others (GPN) to osmotically destabilise it, have provided evidence for the importance of the acidic compartment as a Ca^2±^ store involved in β-cell stimulus secretion coupling [41] as well as in other cell types including neuronal [42] and smooth muscle [43] cells. The specific role for NAADP in accessing these stores has also been shown pharmacologically using competitive inhibition of NAADP-sensing with the putative NAADP-receptor antagonist Ned-19 [17], [20]. The potential role of the two-pore channel as either a direct or indirect component of the NAADP-receptive machinery has also been demonstrated by several groups [44], [45], [46], [47] including ourselves [17], [31].

However, it should be noted that technical differences between studies mean that the current literature from other cells types cannot necessarily be extrapolated directly to the β-cell. Most importantly, pharmacological inhibition may lead to off-target effects. This is especially the case when the function or integrity of an entire organelle is disrupted, as in the case of Bafilomycin A1 or GPN, manoeuvres which have been shown to also block IP_3_- and cADPR-mediated signals in Jurkat T-lymphocytes [48]. Likewise, whole-body deletion of a gene which is expressed in other metabolically relevant tissues could have significant subsequent effects on islet function; the *Tpcn* (and particularly *Tpcn2*) family are particularly strongly expressed in hematopoetic lineages in mice (bioGPS.org) and their deletion from these tissues might conceivably affect innate immunity or alter inflammatory status, with subsequent impacts on islet and β-cell function [49].

We therefore chose in the present study to use (1) targeted gene inactivation, achieved through *Lox*P-mediated recombination of floxed *Tpcn2* alleles, and (2) investigations of β-cell Ca^2±^ responses in whole islets. The latter preparation provides a reliable means of studying robust and reproducible [Ca^2+^]_i_ signals in the absence of changes in β-cell physiology and responsiveness which can accompany islet disruption [26], [27]. Although the Ca^2+^ indicator used, Fluo-8, is not partitioned selectively into β-cells, increases in [Ca^2+^]_i_ in response to glucose (or GLP-1) are likely to be restricted to these cells, and a small number of δ-cells, rather than α-cells which comprise ∼20% of the islet [26].

Examined in this setting, loss of *Tpcn2* exerted no apparent effect on Ca^2+^ signals provoked by nutrients or incretin. Whilst this may seem to question previous findings on *Tpcn2*^*−/−*^ mice, it should be emphasised that our earlier studies involved the use of isolated β-cells [17]. It is therefore possible that the involvement of NAADP in glucose responses may be less susceptible to deletion of *Tpcn2* in the context of the intact islet, given the complex communication and feedback mechanisms existing between β-cells within the micro-organ [50].

In contrast, and despite minimal effects of *Tpcn2* deletion on glucose tolerance, glucose-evoked Ca^2+^ responses and insulin secretion [17], responses to infused NAADP were eliminated in β-cells from whole body *Tpcn2*^*−/−*^ mice. The present data therefore suggest that such currents, activated by high NAADP, are not required for normal Ca^2+^ oscillations in response to glucose or GLP-1 (see Fig. 3, Fig. 4, Fig. 5). However, we would note that the present studies did not explore the impact of NAADP in β*Tpcn2* KO mouse islets and it is conceivable that these NAADP-regulated currents persisted, perhaps as a result of up-regulation of *Tpcn1* (Fig. 1C). Alternatively, smaller currents, which cannot be readily detected by patch clamp electrophysiology, may be critical and contribute to local changes in membrane potential which regulate Ca^2+^ influx.

It is conceivable that the tendency for increases in *Tpcn1* expression after *Tpcn2* deletion, as seen in Fig. 1C is sufficient to compensate for the absence of the latter, especially given that basal levels of *Tpcn1* mRNA in the islet are significantly (>10-fold) higher than those of Tpcn2 [17], as also observed in other tissues such as liver [31]. Correspondingly, *Tpcn1*^*−/−*^ mice show marked defects in both glucose tolerance and in glucose-stimulated insulin secretion. Thus, it is possible that significant functional redundancy exists between TPC1 and TPC2 in the β-cell. Studies using mice deleted for *Tpcn1* alone or both alleles in these cells will be necessary to test this hypothesis.

### 4.2 GLP-1 and Ca^2+^ dynamics

In the physiologically relevant setting of the perifused whole islet, we observed only rather subtle effects of GLP-1 on Ca^2+^ signals induced by low, stimulatory (8 mM) concentrations of glucose (Fig. 5). These observations are thus compatible with earlier studies [25], [26], [27] demonstrating a role for GLP-1 in controlling Ca^2+^ dynamics in β-cells. More noticeable was the complete lack of any observable effect of GLP-1 on Ca^2+^ dynamics induced by both intermediate (11 mM) and elevated (17 mM) glucose concentrations, despite marked increases in insulin secretion under the same conditions (Fig. 4, Fig. 5). This finding suggests that at these high glucose concentrations GLP-1 principally acts by potentiation of glucose-induced insulin secretion and does not involve an increase in electrical activity or [Ca^2+^]_i_. However, at lower glucose concentrations (8 mM), GLP-1 elicited an increase in [Ca^2+^]_i_ that likely reflects stimulation of electrical activity. Thus, the mode of action of GLP-1 differs depending on the glucose concentration. Importantly, elimination of *Tpcn2* had no effect on GLP-1-induced changes in Ca^2+^ dynamics or insulin secretion at any of the glucose concentrations tested, arguing against an absolute requirement for these channels in NAADP-mediated effects.

### 4.3 Overall conclusions

The present studies demonstrate that TPC2 is not absolutely required for normal glucose or incretin responses in pancreatic β-cells. We suggest that there may be significant overlap, and therefore functional redundancy, between two pore channel subtypes (*Tpcn1* and *Tpcn2*), allowing NAADP-dependent actions to persist. The present studies also suggest that the actions of GLP-1 on insulin secretion occur predominantly, though not exclusively, *via* mechanisms which are independent of changes in [Ca^2±^]_i_.

## Figures and Tables

**Table 1:** List of primers. qRT-PCR primers were designed to span an exon boundary in order to prevent interaction with genomic DNA as opposed to cDNA. *Tpcn2* flox primers flank a *lox*P site, producing differential product sizes for *flox*ed and wild type alleles. Excision event primers flank both *lox*P sites allowing for confirmation of the *Cre* excision event.

qRT-PCR	*Tpcn2*	F-ATGATGAAGAAGACCCTGAAGTG R-TGTGCTTCATCCTTCTCACC
	β-actin	F-CGAGTCGCGTCCACCC R-CATCCATGGCGAACTGGTG
Genotyping	*Tpcn2* flox	F-ATTCTGGGCTGCTACTGTGG R-GTTGGTCCTCCTAGGCTGTG
	*Ins1cre*	F-ATGTCCAATTTACTGACCG R-CGCCGCATAACCAGTGAAAC
Excision event		F-CTGCTACTGTGGGTGGTATATGG R-CAATGTTATTTGAACTGATGGCGAG
